# Comprehensive analysis of *LAMC1* expression and prognostic value in kidney renal papillary cell carcinoma and clear cell carcinoma

**DOI:** 10.3389/fmolb.2022.988777

**Published:** 2022-09-16

**Authors:** Jianrong Bai, Axiu Zheng, Yanping Ha, Xiaoqing Xu, Yaping Yu, Yanda Lu, Shaojiang Zheng, Zhihua Shen, Botao Luo, Wei Jie

**Affiliations:** ^1^ Department of Pathology, School of Basic Medicine Sciences, Pathology Diagnosis and Research Center of Affiliated Hospital, Guangdong Medical University, Zhanjiang, China; ^2^ Cancer Institute of Hainan Medical University, Haikou, China; ^3^ Department of Oncology, The First Affiliated Hospital, Hainan Medical University, Haikou, China

**Keywords:** kidney renal papillary cell carcinoma (KIRP), kidney renal clear cell carcinoma (KIRC), LAMC1, expression, prognosis

## Abstract

**Background:** Laminin subunit gamma 1 (LAMC1) protein is associated with tumor cell invasion and metastasis. However, its role in kidney cancer remains unclear. In this work, we sought to probe the expression as well as its carcinogenic mechanisms of *LAMC1* in kidney renal papillary cell carcinoma (KIRP) and kidney renal clear cell carcinoma (KIRC).

**Methods:** Public databases including TIMER, Oncomine, UALCAN, TISIDB, TCGA, Kaplan–Meier plotter, UCSC Xena, cBioPortal, SurvivalMeth, KEGG, GeneMANIA, Metascape, GSCALite and GDSC were adopted, and the expression, clinical pathological correlation, prognostic signatures, dominant factors influencing *LAMC1* expression, DNA methylation levels, gene mutations, copy number variations, functional networks, and drug sensitivity were analyzed. Expression of LAMC1 protein in clinical KIRP and KIRC was validated using tissue array.

**Results:** *LAMC1* expression in KIRP and KIRC were significantly higher than those in normal tissues. High *LAMC1* expression indicated poor overall survival in KIRP patients and better overall survival in KIRC patients. Through the univariate and multivariate Cox analysis, we found that high *LAMC1* expression was a potential independent marker for poor prognosis in KIRP, however it implied a better prognosis in KIRC by univariate Cox analysis. In addition, the *LAMC1* expression in KIRP and KIRC was negatively correlated with methylation levels of *LAMC1* DNA. Interestingly, *LAMC1* expression was positively correlated with the infiltration of CD8^+^ T cells, dendritic cells and neutrophils in KIRP; however, it was positively correlated with the infiltration of CD4^+^ T cells, macrophages and neutrophils but negatively correlated with B cells in KIRC. Moreover, high level of CD8^+^ T cells is beneficial for KIRC prognosis but opposite for KIRP. *LAMC1* may participate in signaling pathways involved in formation of adherens junction and basement membrane in KIRP and KIRC, and the high expression of *LAMC1* is resistant to most drugs or small molecules of the Genomics of Drug Sensitivity in Cancer database.

**Conclusion:** Enhanced *LAMC1* expression suggests a poor prognosis in KIRP while a better prognosis in KIRC, and these opposite prognostic signatures of *LAMC1* may be related to different immune microenvironments.

## Introduction

The morbidity of renal cell carcinoma (RCC) has grown by 2% per year globally over the past 20 years (Ljungberg et al., 2019). In 2020, there were 73,750 new cases and 14,830 deaths of RCC patients reported in the United States (Ljungberg et al., 2019; Siegel et al., 2020). Kidney renal clear cell carcinoma (KIRC) and kidney renal papillary cell carcinoma (KIRP) account for 70%–85% and 10%–15% of all RCC patients, respectively, and KIRC is the most common pathological type of RCC. About 20%–30% of KIRC patients are diagnosed with advanced clinical stages (Escudier, 2007; Oudard et al., 2007). Approximately 30% of KIRC patients develop recurrence and progression despite surgical resection of the primary tumor (Ferlay et al., 2013; Hsieh et al., 2017). For non-metastatic KIRC, the recurrence rate is still as high as 20%–40% after partial or radical nephrectomy (Frank et al., 2003). Furthermore, KIRC is a chemo- and radio-resistant neoplasia and alternative treatment options are limited (Geissler et al., 2015). Clinical practice demonstrated that only a small percentage of patients with KIRC can benefit from targeted therapy and immunotherapy (Topalian et al., 2012; Motzer et al., 2015), and the clinical prognosis and treatment of KIRP are limited. Therefore, the identification of new prognostic and therapeutic biomarkers has important clinical significance.

In clinical practice, cancer biomarkers can be used for the purpose of diagnosis or prognosis in personalized medicine. With the deepening of the understanding of the molecular etiology of RCC, several effective targeted therapies have been applied in clinical treatment, including immunotherapies, and use of multiple kinase inhibitors (Hsieh et al., 2017). However, most RCC patients still die from their diseases because of resistance to these therapies (Linehan and Ricketts, 2014). Current studies on renal cancer biomarkers are mainly focusing on the identification of molecular markers of prognostic signatures and the prediction of the metastatic potential of individual tumors (Tunuguntla and Jorda, 2008; Eichelberg et al., 2009). Cell-matrix adhesion is an important pathological process in the malignant progression of tumor cells. As one of the main components of cell-matrix adhesion molecules, laminin uses the C-terminal LG1-3 domain and the LG4-5 domain as binding sites, connects the extracellular matrix to intracellular components by binding to transmembrane receptors (including integrin receptors and non-integrin receptors), and mediates various signaling (Sonnenberg et al., 1988; Aumailley, 2013). Members of laminin family are composed of three chains named α chain (α1-5), β chain (β1-3) and γ chain (γ1-3). *LAMC1*, which encoding the laminin γ one chain, is widely expressed in the basement membrane and is related to tissue development (Engbring and Kleinman, 2003; Schéele et al., 2007; Gritsenko et al., 2012; Aumailley, 2013). The overexpression of LAMC1 is related to tumor progression and poor prognosis in cancers such as endometrial carcinoma (Kunitomi et al., 2020), hepatocellular carcinoma (Zhang et al., 2017), gastric cancer (Han et al., 2021) and meningioma (Ke et al., 2013), highlighting the significance of molecular targeting LAMC1 in cancer treatment. However, the roles and mechanisms of LAMC1 in RCC remain unclear.

In this investigation, we adopted several publicly accessible databases to analyze *LAMC1* expression and its association with the clinical characteristics and prognosis in KIRP and KIRC. Then, a prognostic signature for KIRP and KIRC patients was constructed. We also focused on the relation of *LAMC1* expression to immune cells infiltration and the immunomodulator-related molecules expression. Furthermore, we explored how *LAMC1* may participate in signaling pathways, biological processes, and drug resistance. Our results revealed the expression status and prognostic signature of *LAMC1* in KIRP and KIRC, and uncovered the impacts of *LAMC1* on immune cell infiltration, and immunomodulator-related molecules in RCC.

## Materials and methods

### Analysis of *LAMC1* gene expression

The Oncomine database (https://www.oncomine.org/), a publicly available microarray database, was used to analyze the mRNA expression level of the LAMC family in different cancers (Rhodes et al., 2007). Tumor tissue was compared with normal controls for the LAMC family members applying *t*-statistics based on the thresholds of *p*-value = 0.0001 and fold change (FC) ≥ 2. The tumor immune estimation resource (TIMER) database (https://cistrome.shinyapps.io/timer/) is a comprehensive resource for the systematic analysis of immune infiltrates across diverse cancer types by using the Wilcoxon test based on the thresholds of *p*-value < 0.05 (Li et al., 2017). The *LAMC1* mRNA and protein expression levels were compared between RCC and normal tissues using the DiffExp module of TIMER. We used the University of Alabama Cancer database (UALCAN, http://ualcan.path.uab.edu/), containing RNA sequences and clinical information from 33 types of tumors to assess the correlation between *LAMC1* gene expression levels and clinicopathological features in KIRP and KIRC patient (Chandrashekar et al., 2017). Differences with a *p*-value < 0.05 were considered statistically significant. The functions and purposes of using various public online databases in this study were detailed in Supplementary Table S1.

### Tissue microarray and immunochemistry staining

The tissue microarray was obtained from Shanghai Outdo Biotech Co.,LTD. Statistical cases include 10 normal kidney tissues and 168 tumor tissues samples (KIRC:138; KIRP: 30). The immunochemistry staining (IHC) was performed using DAKO automatic immunohistochemistry instrument with the programs of “Autostainer Link 48 Usage Guide”. The array was incubated with primary antibodies against LAMC1 (Cat: ABP55085, Abbkine, Wuhan, China) at 1:25 dilution for overnight at 4°C. Antigenic sites were visualized using a DAB kits. The scores of *LAMC1* were calculated as follows: 0, negative; 1, weak; 2, moderate; or 3, strong. The percentage of positive cells was scored as follows: 1, 0–25% positive cells; 2, 26–50% positive cells; and 3, 51–75% positive cells, and 4, 76–100% positive cells. The total immunoreactive scores were determined by nuclear staining score plus cytoplasm membrane staining score, and the IHC scores were determined independently by two pathologists who were blinded to the patients’ clinical data and original pathology reports.

### TCGA data collection and Cox regression

The expression data and mRNA expression profiles of patients with KIRP and KIRC and the clinical information related to survival time were retrieved from Genomic Data Commons data portal of The Cancer Genome Atlas (TCGA, https://portal.gdc.cancer.gov/repository) (KIRP, 321 cases including 32 normal samples; KIRC, 604 cases including 72 normal samples; workflow type, HTSeqCounts) (Weinstein et al., 2013). We used block diagrams to visualize the differences in discrete variable expressions. The HTSeq count data of RNA-Seq gene expression in 289 KIRP and 532 KIRC patients were used for further analysis. Wilcoxon symbolic rank test and logistic regression were used to evaluate the association between clinical factors and *LAMC1* expression. Multivariate Cox regression and Kaplan-Meier methods were used to determine the role of *LAMC1* expression related to the overall survival (OS) of RCC patients and clinical features, including age, gender, grade, stage, characteristics of the primary tumor (T) and distant metastasis (M). The low and high expression groups were distinguished by the median risk score for *LAMC1* expression level as the cutoff value.

### The correlation analysis between *LAMC1* expression and immunity, neoantigen and tumor mutational burden

We evaluated the correlation between *LAMC1* expression in the RCC samples and the six kinds of infiltrating immune cells including B cells, CD4^+^ T cells, CD8^+^ T cells, neutrophils, macrophages, and dendritic cells using “Immune-Gene” module in TIMER2 database. Then we explored the effects of the infiltration immune cell levels on the prognosis of KIRP and KIRC by using the TIMER platform. In addition, the relationship between *LAMC1* expression level and immunoinhibitors and immunostimulators were further studied by the TISIDB database (http://cis.Hku.hk/TISIDB/), a public database for analyzing immune cell and immunoregulatory molecule in different tumors (Ru et al., 2019). The Kaplan–Meier plotter database (http://kmplot.com/analysis/) can be quick and intuitive for prognostic analysis (Nagy et al., 2021), which contains survival data on 54,675 genes from 10,461 cancer samples. We then used this database for prognostic analysis based on *LAMC1* expression levels in related enriched or decreased immune cell subsets including B cells, CD4^+^ memory T cells, CD8^+^ T cells, macrophages, natural killer (NK) T cells, regulatory T (Treg) cells, Type 1 T-helper (Th1) cells, and Type 2 T-helper (Th2) cells (grouping conditions: auto select best cutoff). According to the degree of immune infiltration levels, the ESTIMATE algorithm was used to calculate immune scores, stromal scores and estimate immune scores (the sum of immune score and stromal score) for each tumor sample (Yoshihara et al., 2013). We visualized the correlation between *LAMC1* gene expression and these scores using the R software packages “estimate” and “limma”. Neoantigen encoded by a mutated gene in tumor cells, coming from biological events such as point mutations, deletion mutations, and gene fusions. The number of neoantigens per tumor sample was calculated by SCANNEO algorithm (Wang et al., 2019). Tumor mutational burden (TMB), as a quantifiable biomarker, can be used to reflect the number of mutations contained in a tumor cell, which was visualized with R software packages “ggstatsplot” (Jardim et al., 2021). In addition, Spearman’s rank correlation coefficient was applied to analyze the relationship of *LAMC1* gene expression and tumor immunity, neoantigens and TMB of each tumor sample. These results presented as scatter plots.

### Scoring of anti-cancer immunity

With the widespread use of immune checkpoint blockade agents in clinical practice, tumor immunity has been widely concerned in recent years and has received a good clinical response, pointing out a new direction for the treatment of cancer patients. The Cancer-Immunity Cycle can be roughly divided into seven steps (Chen and Mellman, 2013). These seven steps finely modulate the overall direction of antitumor activity. The scores of anti-cancer immunity were calculated by using ssGSEA algorithm with R package “GSEABase” based on specific gene set. The median value of *LAMC1* were used as the cutoff value and our cohort were divided into high expression group and low expression. These results were presented with boxplot with the assistance of online web-Sangerbox 3.0 (http://vip.sangerbox.com/home.html) with *t-*test.

### Analysis of copy number variation and DNA methylation

To investigate the possible factors influencing *LAMC1* expression, the California Santa Cruz Cancer Genomics Browser (UCSC Xena, http://xena.ucsc.edu/) database was used (Goldman et al., 2020). In addition, to confirm the prognostic value of *LAMC1* methylation and copy number variation (CNV) in KIRP and KIRC, UCSC Xena databases was searched to investigate the effects of *LAMC1* methylation and CNV on OS. The alteration frequency and CNV of the *LAMC1* gene was also analyzed via the cBioPortal database (http://www.cbioportal.org/). We used SurvivalMeth database (http://bio-bigdata.hrbmu.edu.cn/survivalmeth/) to study the differences in *LAMC1* DNA methylation in region of whole gene between normal kidney tissues and KIRP and KIRC tissues (Method: *t*-test, Threshold Value: 0.01, Grouping Strategy: Maxstate) (Zhang et al., 2021).

### Pathway, Co-expression network, and functional enrichment analyses

To explore whether *LAMC1* gene and a set of genomes with the highest correlation are differentially expressed (high or low groups were distinguished by the median value of *LAMC1* expression level), we used GSEA algorithm analysis (https://www.gsea-msigdb.org/gsea/index.jsp) based on TCGA data of KIRP and KIRC (Subramanian et al., 2005). Gene sets with *p*-value < 0.05 and false discovery rate (FDR) Q-value < 0.25 were considered the thresholds. The results of gene enrichment analysis were plotted using R packages such as “ggplot2” and “grid” in R software (https://www.R-project.org, Version 4.0.4). We obtained the gene interacting with *LAMC1* through the GeneMANIA network (http://genemania.org/), which could establish genetic interactions, protein–DNA interactions, and protein–protein interactions (PPI) (Warde-Farley et al., 2010). When the gene mane “*LAMC1*” was typed in the search interface, GeneMANIA automatically searches related public databases to establish a co-expression network. In addition, we carried out Gene Ontology (GO) and Kyoto Encyclopedia of Genes and Genomes (KEGG) functional enrichment analysis of the interacting genes using the Metascape portal (http://metascape.org/gp/index.html) (Zhou et al., 2019).

### Gene set enrichment and drug resistance analysis

GSCALite database (http://bioinfo.life.hust.edu.cn/web/GSCALite/) offers multiple types of cancer gene set analyses, including mRNA expression, single nucleotide variation (SNV), methylation, cancer-related pathways, and miRNA networks (Liu et al., 2018). We analyzed the effect of *LAMC1* in cancer-related signaling, the expression of some genes of interest, and the miRNA network between them. In addition, we analyzed the correlation between *LAMC1* expression and drug sensitivity based on the Genomics of Drug Sensitivity in Cancer (GDSC) database by Spearman correlation analysis. If the correlation result is positive, the high expression of this gene is associated with specific drug resistance. Drug module correlation analyses for all cancer cell lines and other analyses were performed using the KIRP and KIRC TCGA dataset.

### Statistical analysis

All statistical analyses were performed using R software (Version 4.0.4). Receiver operating characteristic (ROC) curves were established to evaluate the diagnostic significance of *LAMC1* expression using the “pROC” package of R, and the area under the ROC curve (AUC) indicated the magnitude of diagnostic efficiency. AUC >0.7 indicated good accuracy. Unpaired and paired Student *t*-test were performed to analyze the statistical difference of *LAMC1* gene expression in normal and tumor tissues. The associations between clinical features and *LAMC1* expression were evaluated using the Wilcoxon signed-rank test and logistic regression. Clinical features related to overall survival (OS) in KIRP and KIRC patients were identified using Cox regression and the Kaplan-Meier method. Univariate and multivariate Cox analyses were used to explore the independent prognostic significance of *LAMC1* expression level and clinical features on OS in KIRP and KIRC patients. The correlations of *LAMC1* expression with immune cells infiltration were evaluated using Spearman’s correlation analysis. The thresholds were referenced the related methods section. All *p*-values were adjusted by false discovery rate (FDR) calculated using the Benjamini–Hochberg method, and 5% FDR (q-value <0.05) was set as the threshold.

## Results

### Expression levels of *LAMC* gene family in kidney cancer patients

**FIGURE 1:**
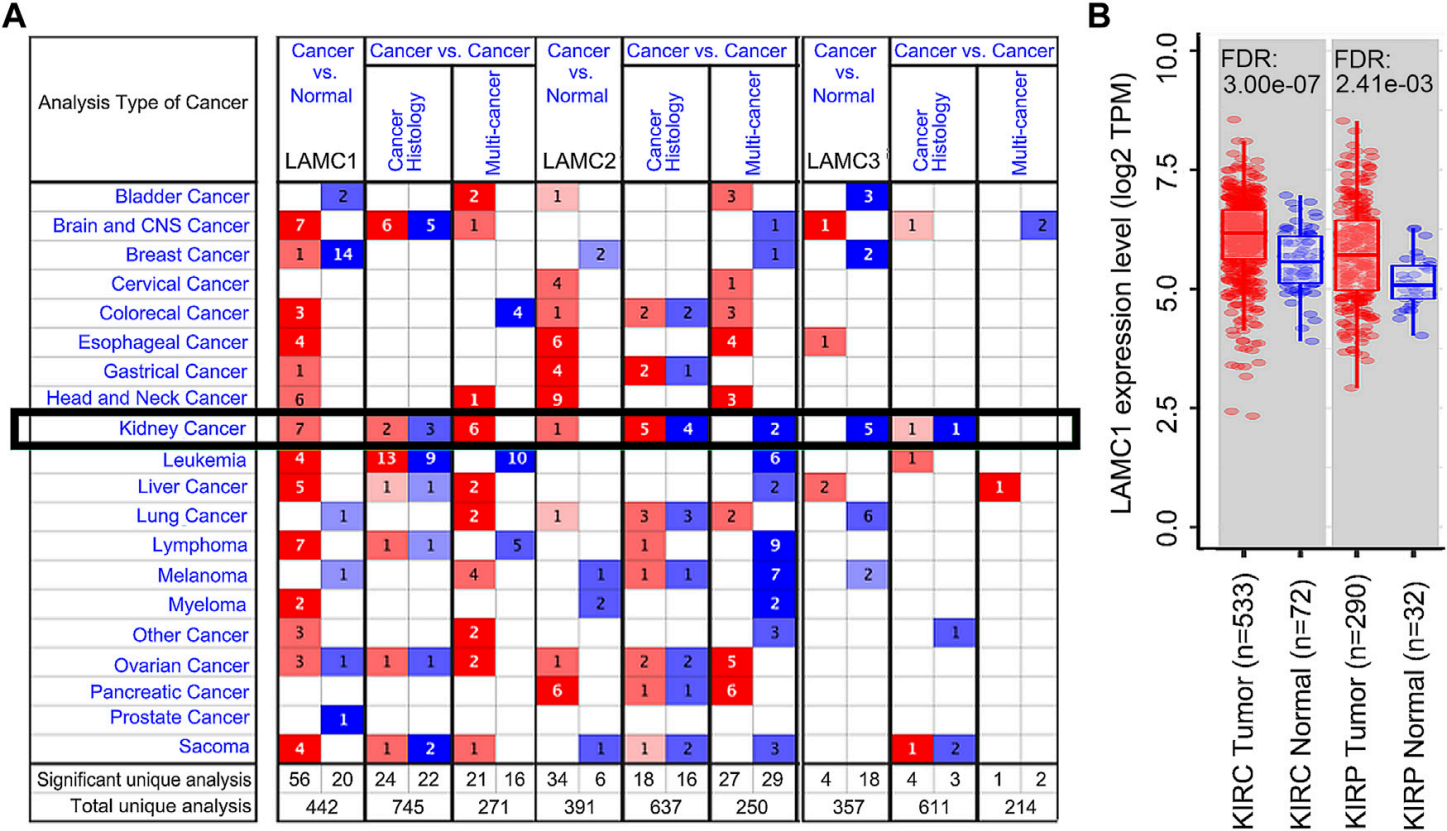
The expression levels of *LAMC1* across human cancers at mRNA level. **(A)** The expression levels of Laminin C (*LAMC*) family members in different types of cancers based on Oncomine database. The number in each cell represents significance datasets of genes up-regulated or down-regulated in a particular cancer. Red box represents high expression in tumors, blue box represents low expression in tumors and white box represents no difference in tumors and normal tissues. **(B)** Differential expression levels of *LAMC1* in KIRP and KIRC between tumor groups and normal groups based on TIMER database.

**FIGURE 2:**
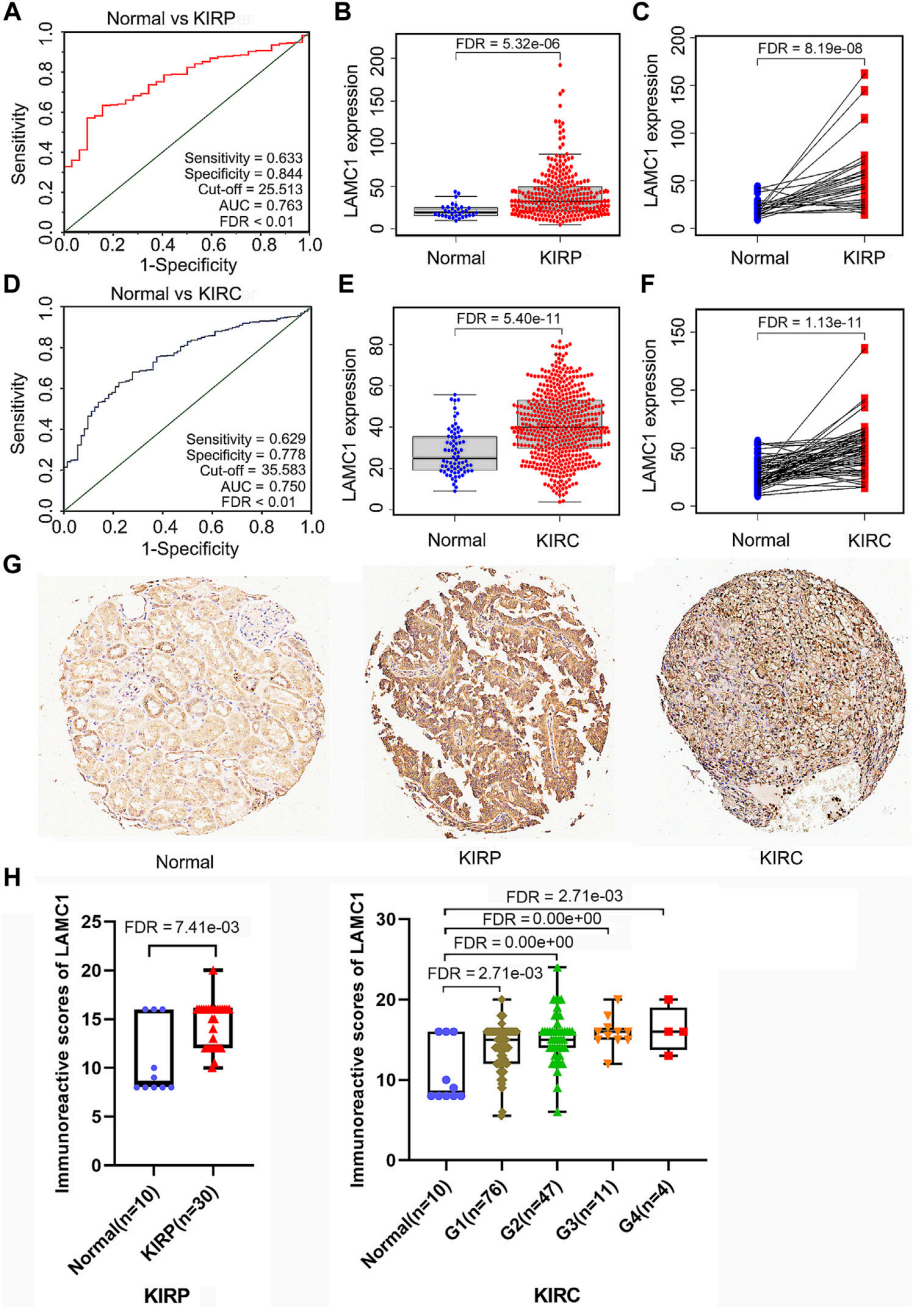
*LAMC1* mRNA and protein expression levels in two types of renal carcinoma. **(A,D)** The receiver operating characteristic (ROC) curve for *LAMC1* expression in normal kidney tissue and kidney cancer was built based on data downloaded from TCGA database. **(B–C,E–F)** The mRNA level of *LAMC1* in kidney cancer and normal tissues. **(B–C)**, KIRP. **(B)**: N: T = 32:289; **(C)** N: T = 31:31, **(E–F)**, KIRC; **(E)** N: T = 72:532, **(F)** N: T = 72:72; The figure represents the number of the normal(N) or tumor(T) cases; **B** and **E**, unpaired *t*-test; **C** and **F**, paired *t*-test. **(G)** Three representative images of tissue microarray results are used here. Positive immunostaining was located in the nucleus or cytoplasm. Validation of protein expression of *LAMC1* in kidney cancer and normal tissues based on tissue microarray staining data. **(H)** The immunoreactive score of LAMC1 IHC staining presented by boxplot with Student’s *t* test or one-way analysis of variance (ANOVA).

**FIGURE 3:**
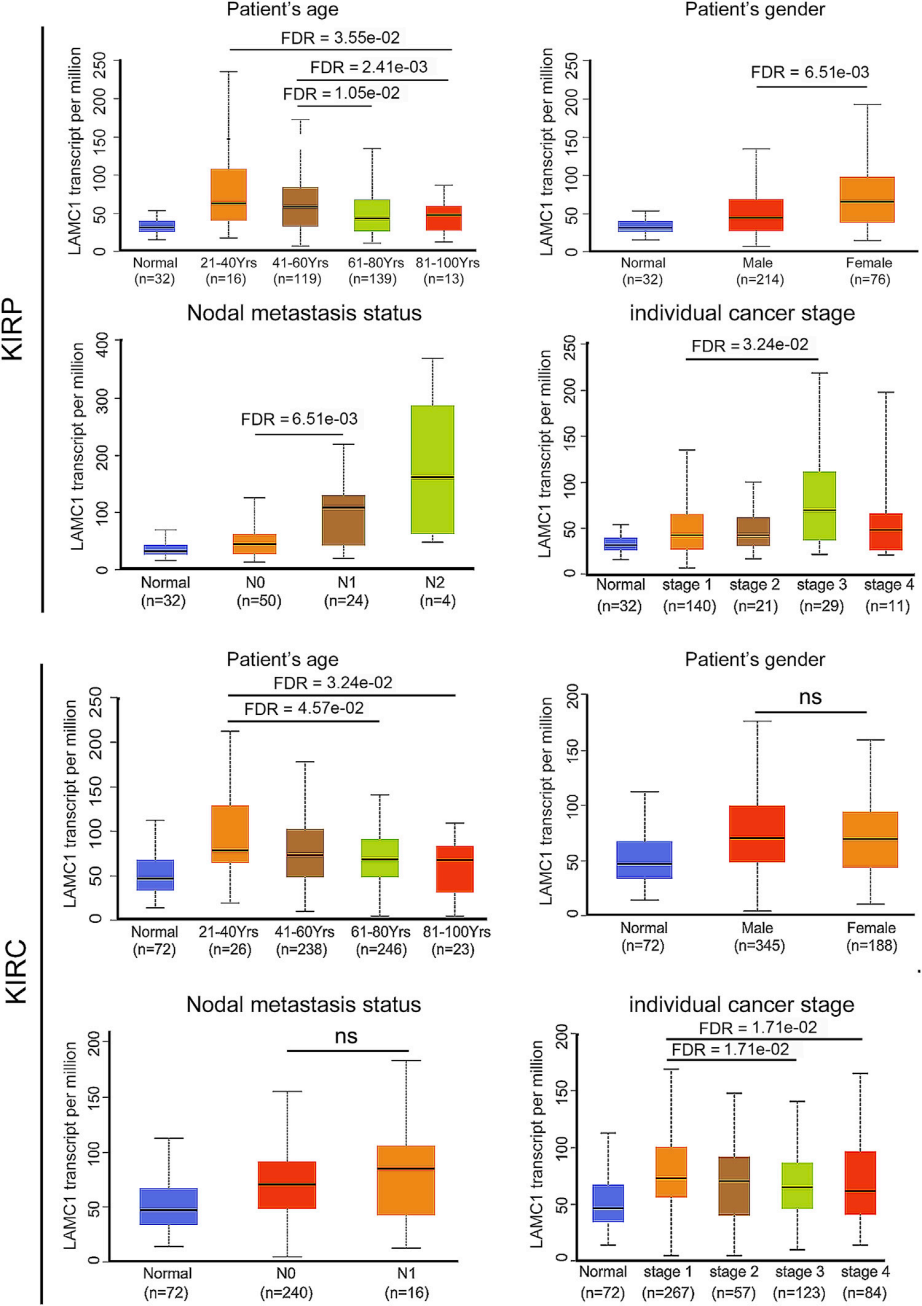
The relation of *LAMC1* expression to the clinicopathological features including age, gender, nodal metastasis status, and individual cancer stage in KIRP and KIRC.

Using the Oncomine database, we compared the differential expression levels of *LAMC* family members between cancers and its related normal tissues. Of the three members of the *LAMC* family, *LAMC1* and *LAMC2* were upregulated in kidney cancers, while *LAMC3* was downregulated in kidney cancers (Figure 1A). The significant changes to the expression of the *LAMC* family in different types of kidney cancer and normal kidney tissue are detailed in Supplementary Table S2. Furthermore, expression of *LAMC1* mRNA was found to be significantly higher in KIRP and KIRC samples based on the TIMER database (FDR <0.01, Figure 1B). *LAMC1* mRNA was also upregulated in many other types of tumors besides KIRP and KIRC (Supplementary Figure S1). We further used TCGA database to analyze the expression profile of *LAMC1* in KIRP and KIRC. Our results suggested that the data were of high quality, as the area under the ROC curve was 0.763 (95% CI, 69.5%–83.2%, FDR <0.01) for KIRP (Figure 2A) and 0.750 (95% CI, 69.7%–80.2%, FDR <0.01) for KIRC (Figure 2D). There was significantly enhanced *LAMC1* expression in KIRP compared with normal tissues (FDR = 5.32e-06, Figure 2B), and the result for paired samples also supported this trend (FDR = 8.19e-08, Figure 2C). Similarly, as shown in Figures 2E,F, higher *LAMC1* mRNA expression was also found in KIRC compared with normal tissues (FDR <0.001). We used tissue microarray to validate the LAMC1 protein expression in clinical samples. Three representative images of tissue microarray results are shown in Figure 2G. The analysis of IHC staining data showed that LAMC1 was positively expressed in the nucleus or cytoplasm in renal cancer tissues, and LAMC1 protein staining was stronger in KIRP and KIRC tissues compared with normal renal tissues, indicating higher expression of LAMC1 protein in RCC servers certain pathophysiological role (Figures 2G,H). Additionally, Figure 2H showed that the total immunoreactive scores for different tumor grades in KIRC. We consequently explored the expression of *LAMC1* in different clinicopathological parameters of KIRP and KIRC, including age, gender, nodal metastasis status, and cancer stage, based on the UALCAN database. As shown in Figure 3, patients that showed higher *LAMC1* expression in KIRP were 20–40 years old, female, of advanced nodal metastasis status and advanced stages of cancer (stage 3–4). Patients that showed higher *LAMC1* expression in KIRC were 20–40 years old and in the early stages of cancer (stage 1–2).

### Survival outcomes and multivariate analysis

**FIGURE 4:**
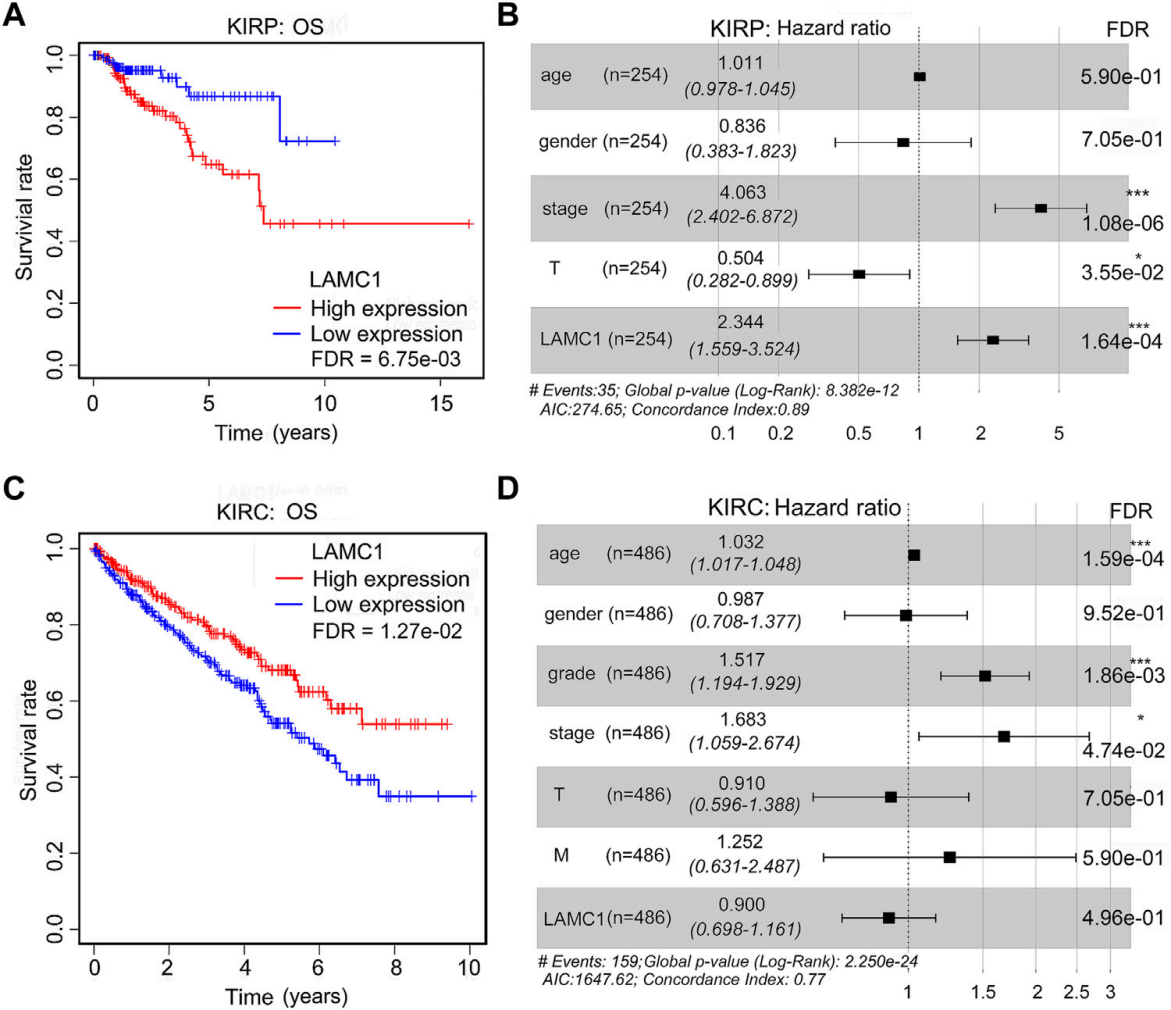
The prognostic signatures of *LAMC1* and clinical parameters in KIRP and KIRC patients. Correlation of different expression of *LAMC1* with survival (OS) of KIRP **(A)** and KIRC **(C)** patients. Survival data were analyzed using Kaplan-Meier method. High or low *LAMC1* expression level was determined in relation to its median expression value. The prognostic signatures of *LAMC1* expression and clinical parameters in KIRP**(B)** and KIRC**(D)** patients in the multivariate Cox analysis model presented as the forest plots.

TCGA database was retrieved for further survival analysis. High expression of *LAMC1* was closely associated with poor OS of KIRP patients (FDR = 6.75e-03, Figure 4A) and better OS of KIRC patients (FDR = 1.27e-02, Figure 4C). According to the “survival” package of R calculation, the five-year survival rate of KIRP and KIRC patients with high expression of *LAMC1* was 64.8% and 66.8%, respectively, and the five-year survival rate of KIRP and KIRC patients with low expression of *LAMC1* was 86.8% and 52.9%, respectively (Supplementary Table S3). Using univariate and multivariate Cox analysis, the prognostic signatures of *LAMC1* and other clinical parameters for KIRP and KIRC were analyzed (Supplementary Table S4). The prognostic signatures of *LAMC1* and other clinical parameters in the multivariate Cox analysis model were presented as the forest plots (Figures 4B,D). For KIRP, the *LAMC1* expression level and stage were independent prognostic indicators in both univariate and multivariate Cox analysis models. Considering that the HR values of T classification fluctuates greatly in univariate and multivariate Cox analysis, we did not consider it to be statistically significant in KIRP. For KIRC, age, stage, and grade were the independent prognostic indicators in both univariate and multivariate Cox analysis models, and *LAMC1* lost its independent prognostic signature in KIRC in the multivariate Cox analysis model.

### Correlation of *LAMC1* expression, immune infiltration and survival in RCC

**FIGURE 5:**
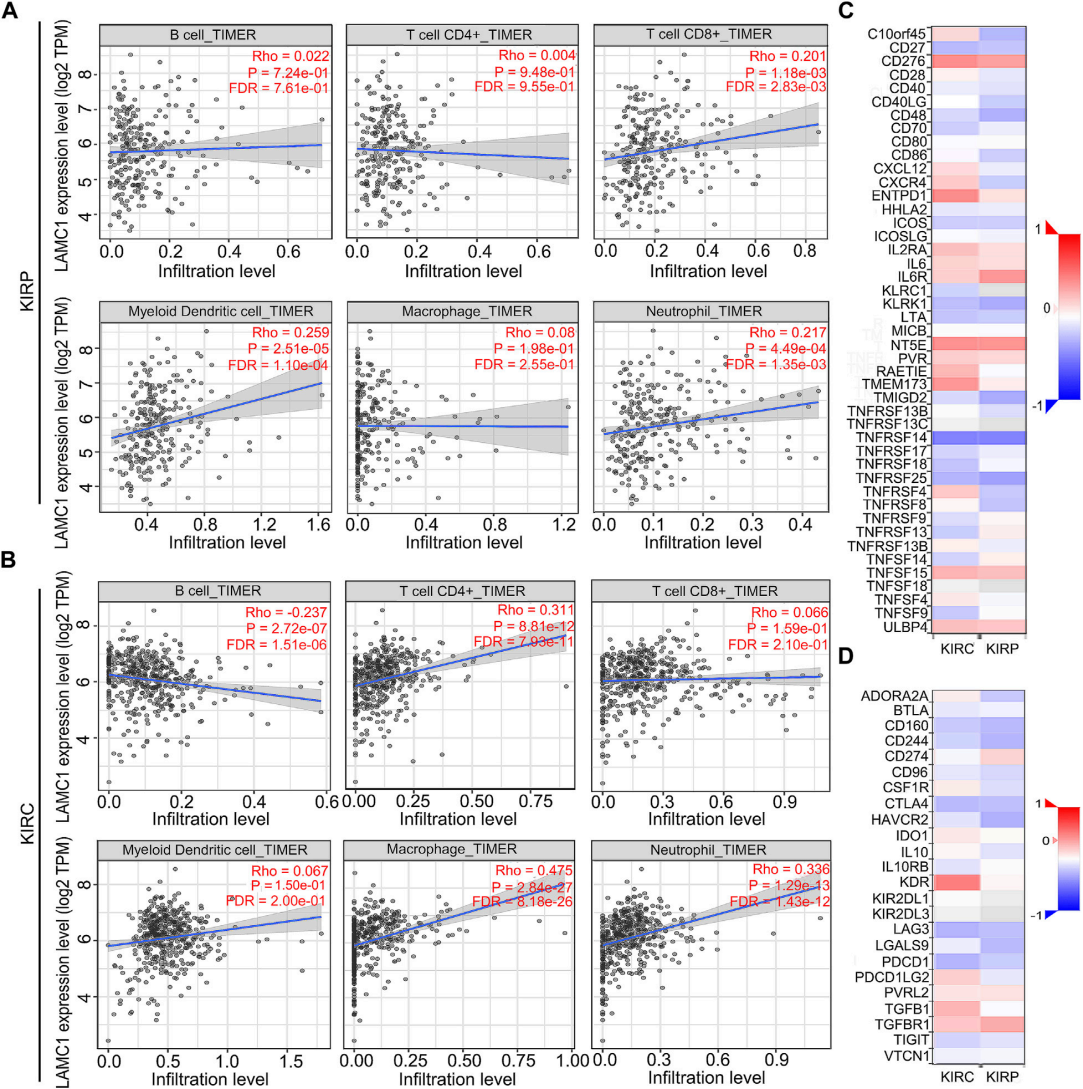
Correlation analysis of *LAMC1* expression and immune cell infiltration levels and immunoregulators in KIRP and KIRC. **(A,B)** Correlation between *LAMC1* expression and tumor immune cells in KIRP and KIRC samples identified using the TIMER algorithm. **(C)** Relationships between expression of *LAMC1* and 45 types of immunostimulators in human KIRP and KIRC using TISIDB database. Red color indicates positively related and blue color indicates the negatively related immunostimulators. **(D)** Relationships between expression of *LAMC1* and 24 types of immunoinhibitors in human KIRP and KIRC samples identified using TISIDB database. Red color indicates positively related and blue color indicates the negatively related immunoinhibitors.

Considering that tumor-infiltrating immune cells (TIICs) are potential therapeutic targets for cancer treatment progression (Sanmamed and Chen, 2018), we thus aimed to determine the composition of TIICs in RCC and further reveal the prognostic values. We used the TIMER database to analyze the correlation of *LAMC1* level with immune cell infiltration levels in RCC. For KIRP, *LAMC1* expression was positively correlated with CD8^+^ T cells (R = 0.201, FDR = 2.83e-03), myeloid dendritic cells (R = 0.259, FDR = 1.10e-04) and neutrophils (R = 0.217, FDR = 1.35e-03) (Figure 5A). For KIRC, the *LAMC1* level showed a positive correlation with infiltrating levels of CD4^+^ T cells (R = 0.311, FDR = 7.93e-11), macrophages (R = 0.475, FDR = 8.18e-26), and neutrophils (R = 0.336, FDR = 1.43e-12) and a negative correlation with B cells (R = −0.237, FDR = 1.51e-06) (Figure 5B). Similarly, the correlation between *LAMC1* and 45 immunostimulators in RCC is shown in Figure 5C, and the correlation between *LAMC1* and 24 immunoinhibitors in RCC is shown in Figure 5D. We noticed some immunomodulator-related genes with strong or significantly differential correlation with *LAMC1* expression, including *TGFB1*, *CD276*, *NT5E* and *KDR*. We used the GSCALite database to further explore the expression and prognosis of *LAMC1* and above genes, and provided a potential miRNA regulatory mechanism for gene expression (Supplementary Figure S2, Supporting file 6). Predicted by database analysis, the miR-29 and miR-200 families are prominent in targeting the above-mentioned gene expression. Since microRNAs play important roles in cancer progression, follow-up experimental validation is still required.

### Prognostic potential of *LAMC1* expression in RCC based on immune cell infiltration

**FIGURE 6:**
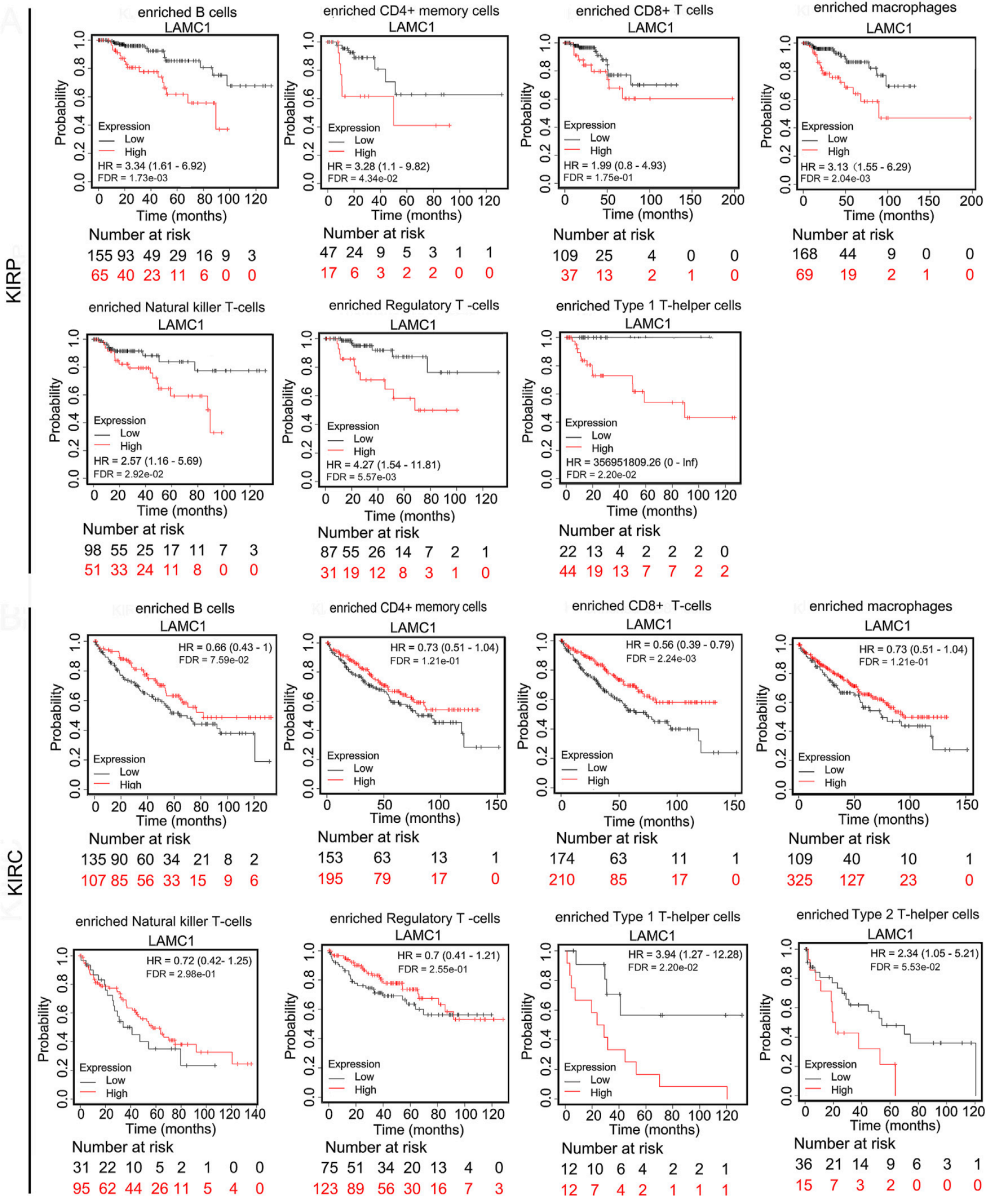
Kaplan–Meier survival curves for RCC patients with differential *LAMC1* expression were constructed based on immune cells enrichment in RCC tumors.

**FIGURE 7:**
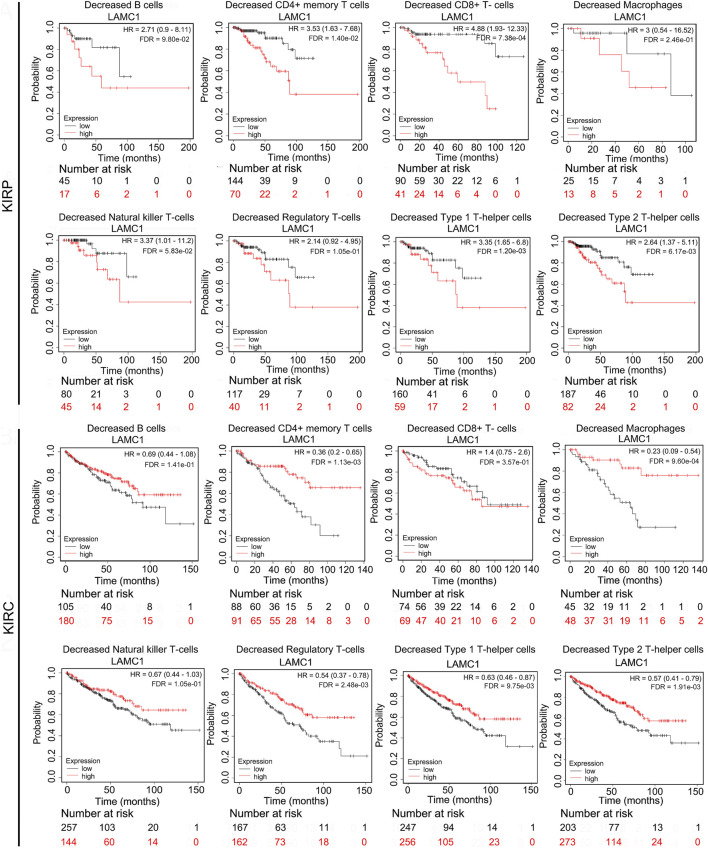
Kaplan–Meier survival curves for RCC patients with differential *LAMC1* expression were constructed based on immune cells depletion in RCC tumors.

Given that the *LAMC1* levels are related to TIICs in KIRP and KIRC (Figures 5A,B), we speculated that *LAMC1* may affect the prognosis of KIRP and KIRC patients partly through mediating immune cell infiltration. We examined the prognostic value of tumor infiltrating immune cells in KIRP and KIRC using Cox proportional hazard model by TIMER. The results indicated that B cells (HR = 378.414, FDR = 2.92e-02) and CD8^+^ T cells (HR = 275289.087, FDR = 0.00) were significantly correlated with clinical prognosis in KIRP (Table 1). Besides, CD8^+^ T cells (HR = 0.143, FDR = 2.34e-02) and Macrophage (HR = 0.006, FDR = 2.65e-02) were significantly correlated with clinical prognosis in KIRC (Table 1). Kaplan–Meier survival curves for RCC patients with differential *LAMC1* expression were constructed based on immune cells enrichment (Figure 6) or decrease (Figure 7). As shown in Figure 6, high *LAMC1* levels in the KIRP cohorts enriched with B cells (HR = 3.34, FDR = 1.73e-03), CD4^+^ memory T cells (HR = 3.28, FDR = 4.34e-02), macrophages (HR = 3.13, FDR = 2.04e-03), NK T cells (HR = 2.57, FDR = 2.92e-02), Treg cells (HR = 4.27, FDR = 5.57e-03), and Th1 cells (FDR = 2.20e-02) had a poor OS. Surprisingly, high expression of *LAMC1* had a poor OS in KIRC enriched with Th1 cells (HR = 3.94, FDR = 2.20e-02), but a better OS in CD8^+^ T cells (HR = 0.56, FDR = 2.24e-03). Similarly, as shown in Figure 7, high *LAMC1* expression in KIRP had a poor OS in the cohorts decreased with CD4^+^ memory T cells (HR = 3.53, FDR = 1.40e-02), CD8^+^ T cells (HR = 4.88, FDR = 7.38e-04), Th1 cells (HR = 3.35, FDR = 1.20e-03) and Th2 cells (HR = 2.64, FDR = 6.17e-03). However, high *LAMC1* expression in KIRC had a better OS in the cohorts decreased with CD4^+^ memory T cells (HR = 0.36, FDR = 1.13e-03), macrophages (HR = 0.23, FDR = 9.60e-04), Treg cells (HR = 0.54, FDR = 2.48e-03), Th1 cells (HR = 0.63, FDR = 9.75e-03) and Th2 cells (HR = 0.57, FDR = 1.91e-03). These results supported our prediction that a high *LAMC1* expression level in KIRP and KIRC affected prognosis partly because of the different TIIC infiltration levels.

**TABLE 1:** The Cox proportional hazard model of six tumor-infiltrating immune cells in KIRP and KIRC.

Cell type	KIRP				KIRC			
	Coefficient	HR	95% CI	FDR value	Coefficient	HR	95% CI	FDR value
B cell	5.94	378.41	3.01–47528.65	**2.92e-02**	−0.89	0.41	0.02–9.31	6.34e-01
CD8+ T cell	12.53	275289.09	1212.18–62518734.86	**0.00e+00**	−1.95	0.14	0.03–0.66	**2.34e-02**
CD4+ T cell	6.04	419.18	0.27–641305.31	1.48e-01	−0.18	0.84	0.06–11.01	9.20e-01
Macrophage	−3.99	0.02	0.00–2.89	1.66e-01	−2.87	0.06	0.01–0.57	**2.65e-02**
Neutrophil	−3.95	0.02	0.00–7620.69	6.12e-02	4.17	64.50	1.32–3147.94	5.82e-02
Dendritic	−4.47	0.01	0.00–0.78	6.01e-02	1.56	4.73	0.85–26.37	1.12e-01

Bold texts indicate statistically significant according to threshold

### The relationships between *LAMC1* expression and immunity, neoantigen and TMB

**FIGURE 8:**
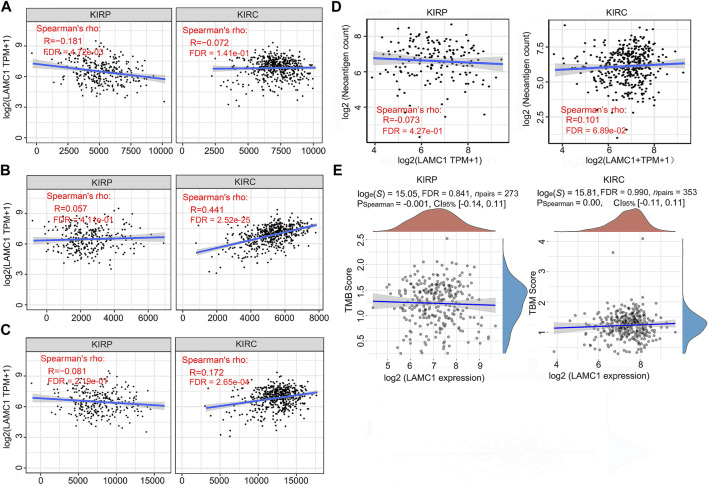
The relationships between *LAMC1* expression and tumor immune microenvironment, neoantigens appearance and TMB. **(A)** Correlation analysis between *LAMC1* expression and immune scores in KIRP and KIRC. **(B)** Correlation analysis between *LAMC1* expression and stromal scores in KIRP and KIRC. **(C)** Correlation analysis between *LAMC1* expression and estimate immune scores in KIRP and KIRC. **(D)** Correlation analysis between *LAMC1* expression and the number of neoantigens in KIRP and KIRC. **(E)** Correlation analysis between *LAMC1* expression and TMB scores in KIRP and KIRC. TMB, Tumor mutational burden.

To further evaluate association of *LAMC1* and immune microenvironment in RCC, we analyzed the relation of *LAMC1* expression to the Cancer-Immunity Cycle, immune neoantigens appearance and tumor mutational burden (TMB). The activities of Cancer-Immunity Cycle can be roughly divided into seven steps. Our results showed that most of activities of Cancer-Immunity Cycle were higher in high *LAMC1* expression groups in KIRC and only just a few steps showed higher immunoactivity in KIRP (Supplementary Figure S3, Supporting file 7). In addition, the ability to recruit CD8^+^ T cells was significantly enhanced in the KIRC group with high *LAMC1* gene expression. We used GSEABase analysis to evaluate immune, stromal and estimates scores in two types of RCC, depending on *LAMC1* expression. Then we found *LAMC1* gene expression had a weak negative correlation with immune scores in KIRP (R = −0.181, FDR = 4.72e-03) and a moderate positive correlation with stromal scores in KIRC (R = 0.441, FDR = 2.52e-25) (Figures 8A,B). The estimate immune scores also showed a positive correlation in KIRC (R = 0.172, FDR = 2.65e-04) (Figure 8C). Then, we performed the analysis for the association of *LAMC1* expression and the number of immune neoantigens, which showed a weak positive correlation in KIRC (R = 0.101, FDR = 6.89e-02) (Figure 8D). However, *LAMC1* gene expression had no significant correlation with TMB in both types of renal cancer (Figure 8E).

### Mutation, CNV and methylation analysis of *LAMC1* gene

**FIGURE 9:**
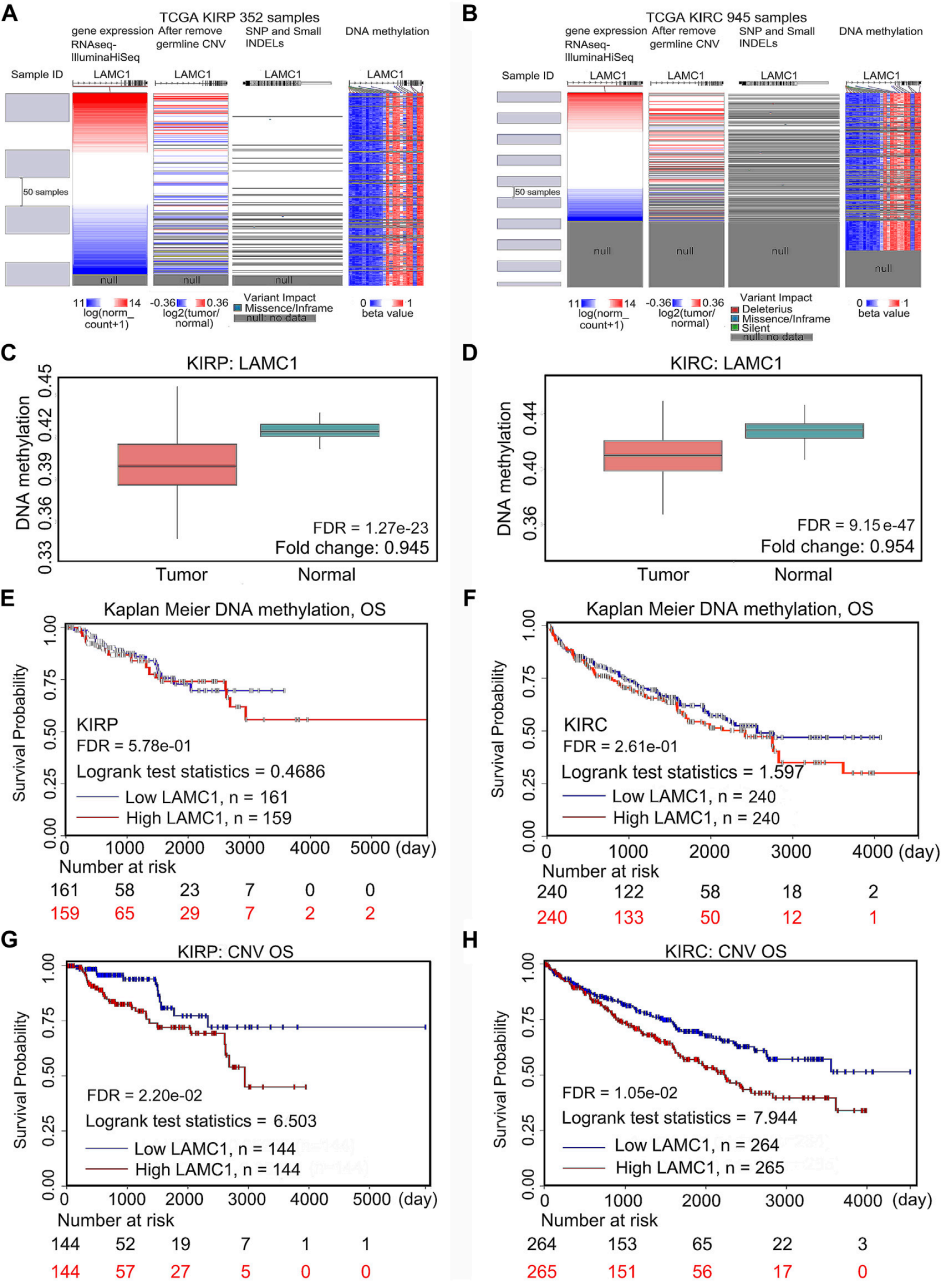
Mutation, CNV, and methylation of *LAMC1* and prognostic value of the *LAMC1* gene expression. **(A,B)** Heatmap showing the correlations between *LAMC1* mRNA and somatic mutations, CNV, and methylation in KIRP **(A)** and KIRC **(B)** using UCSC Xena database. **(C,D)** Comparison of *LAMC1* DNA methylation between normal kidney tissues and KIRP **(C)** and KIRC **(D)** tissues using survivalMeth database. **(E,F)** Relationship between *LAMC1* DNA methylation and OS in KIRP **(E)** and KIRC**(F)** using UCSC Xena. **(G,H)** Relationship between *LAMC1* CNV and OS in KIRP**(G)** and KIRC**(H)** using UCSC Xena. OS: overall survival; CNV: copy number variation.

To assess the cause of elevated *LAMC1* levels in KIRP and KIRC, we used the cBioPortal, UCSC Xena and SurvivalMeth databases to probe the *LAMC1* methylation level, mutations, and CNV status. The results from the cBioPortal dataset showed that *LAMC1* expression was negatively correlated with methylation in KIRP (R = −0.22, FDR = 9.63e-04) and KIRC (R = −0.31, FDR = 1.31e-06) (Supplementary Figure S4A,B, Supporting file 8). Among the subgroups with different CNV, diploid was the dominant type for both KIRP and KIRC (Supplementary Figure S4C,D, Supporting file 8). We studied 831 samples from TCGA database and showed that the mutation rate of *LAMC1* in KIRP and KIRC was very low (<1%) (Supplementary Figure S4E, Supporting file 8). Heat map of *LAMC1* mRNA expression, methylation and copy number in patients with RCC and normal tissues were showed in Figures 9A,B. We found that *LAMC1* DNA was only locally methylated. Even that the correlation between *LAMC1* expression and methylation may be influenced by few outliers (Supplementary Figure S4B, Supporting file 8), the results of SurvivalMeth database further displayed the lower methylation level of *LAMC1* in both KIRP and KIRC (FDR <0.001, Figures 9C,D). Therefore, we concluded that DNA methylation of *LAMC1* was reduced in KIRP and KIRC tissues compared with that in normal tissues. According to UCSC Xena database, methylation of *LAMC1* was not associated with OS prognosis of KIRP and KIRC (FDR >0.1, Figures 9E,F), while high CNV of *LAMC1* indicated poor OS in both KIRP and KIRC (FRD <0.05, Figures 9G,H).

### *LAMC1*-associated signaling pathways, Co-expression network, functional enrichment, and drug sensitivity in RCC

**FIGURE 10:**
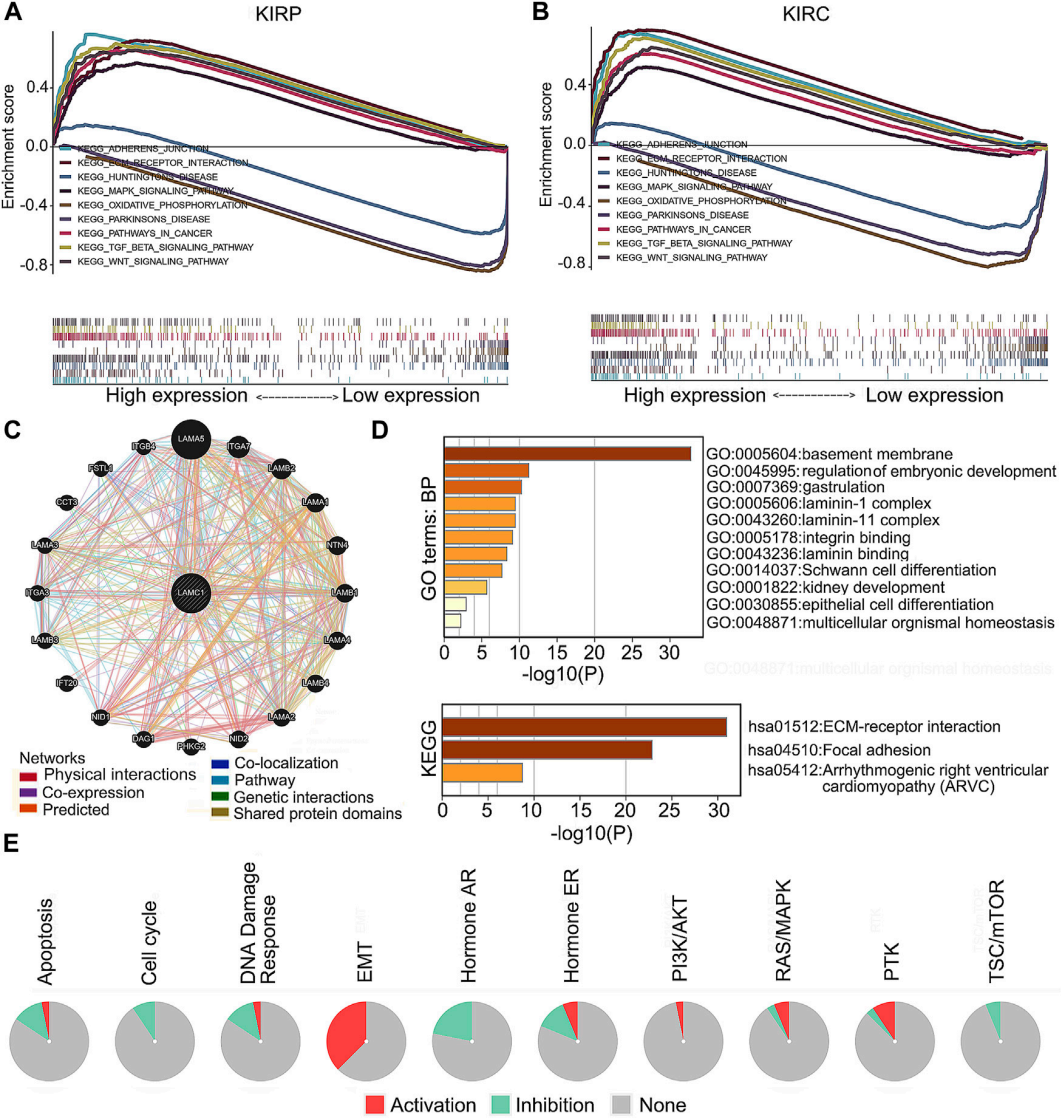
*LAMC1*-associated signaling pathways, co-expression network and functional enrichment. **(A,B)** A pathway enrichment analysis of a rank-ordered gene list using the GSEA software for the high and low *LAMC1* expression in KIRP **(A)** and KIRC **(B)**. **(C)** The co-expression network of *LAMC1* constructed by GeneMANIA. The node size represents the strength of interactions, and the line color represents the types of interactions. **(D)** Effect of *LAMC1* on the biological processes. The histograms show the main biological processes in which *LAMC1* interacting genes (as predicted by the GO and KEGG enrichment analyses) are involved, constructed using the Metascape portal (enrichment conditions: min overlap, three; *p*-value cutoff, 0.01; min enrichment, 1.5). **(E)** Effect of *LAMC1* on the key pathways in cancers detected using GSCALite database. Red represents promotion; green represents inhibition.

**FIGURE 11:**
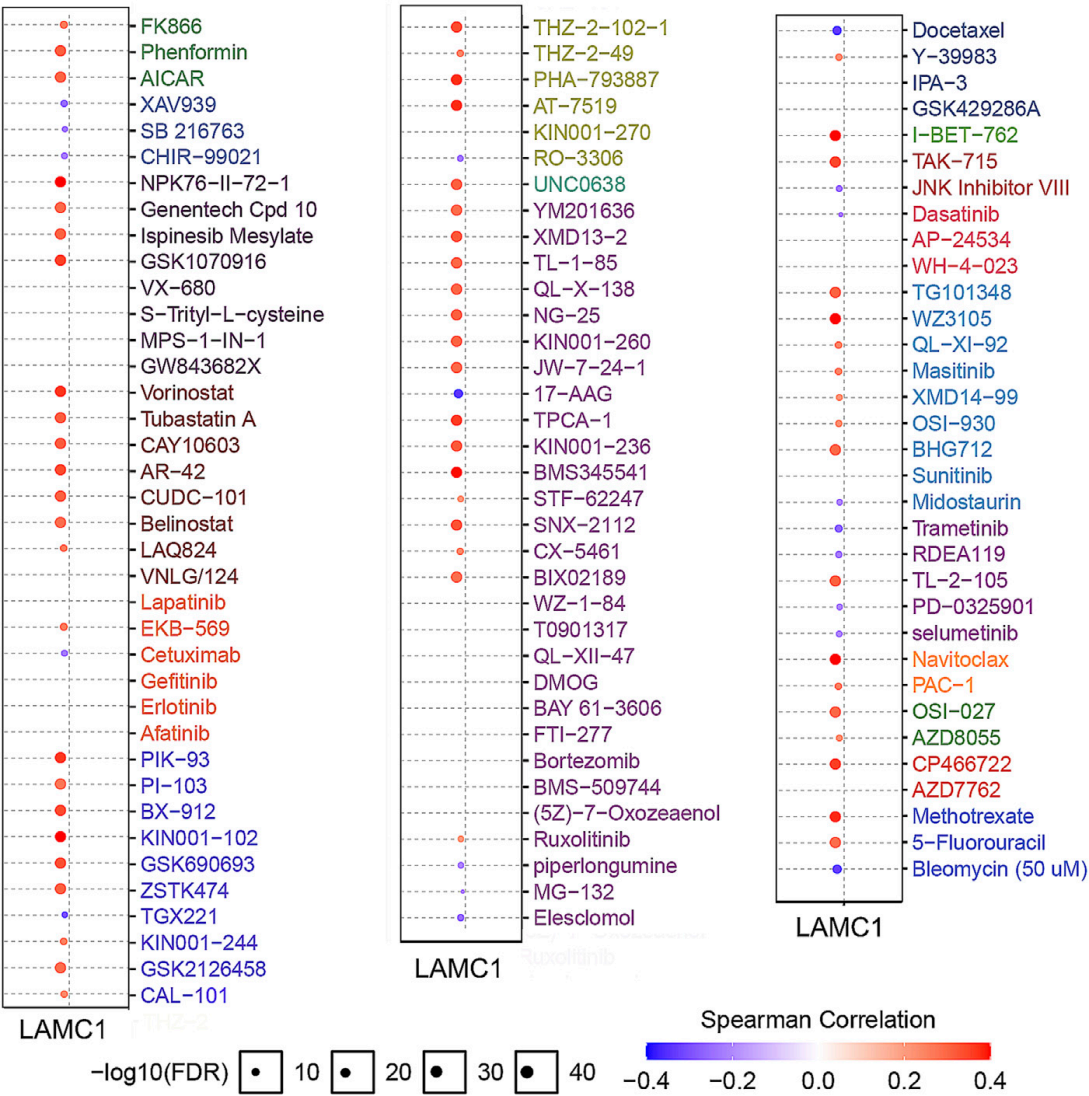
Analysis of drug resistance based on IC50 drug data from the GDSC database (GSCALite). A positive Spearman correlation (red) means that high gene expression correlates with drug resistance, a negative Spearman correlation (blue) means that low gene expression correlates with drug resistance.

To screen for differentially activated signaling pathways in KIRP and KIRC, we compared high and low *LAMC1* expression datasets by GSEA analysis. According to the normalized enrichment scores, significantly enriched signaling pathways were identified. Adherens junctions, extracellular matrix receptor interaction, the MAPK (mitogen-activated protein kinase) signaling pathway, the TGF-β (transforming growth factor beta) signaling pathway, and the Wnt signaling pathway were differentially associated with the high *LAMC1* expression phenotype. At the same time, gene sets related to oxidative phosphorylation, Huntington’s disease, and Parkinson’s disease were differentially associated with the low *LAMC1* expression phenotype (Figures 10A,B). In addition, the functional networks between *LAMC1* and other genes were assessed by GeneMANIA, and LAMA5 displayed the most complex connection with *LAMC1* (Figure 10C). Additionally, biological processes (BP) and pathways of *LAMC1*-interacting genes enriched in GO and KEGG were evaluated by Metascape. We found that the basement membrane formation was the most significantly enriched BP, and signaling initiated by ECM–receptor interaction and focal adhesions formation were the most significant pathways (Figure 10D). We also evaluated the role of *LAMC1* in the activity of cancer-related pathways and drug sensitivity in RCC by GSCALite. We found that the epithelial–mesenchymal transition (EMT) process was mainly activated while Hormone AR signaling pathway was most inhibited in RCC (Figure 10E). Finally, when considering the drug sensitivity associated with *LAMC1* expression, RCCs with high *LAMC1* expression were resistant to sets of drugs or small molecules such as phenformin, NPK76-II-72-1, vorinostat, and PIK93, whereas RCCs with lower levels of *LAMC1* expression were resistant to small sets of drugs or small molecules including XAV939, 17-AAG, docetaxel, and bleomycin (Figure 11).

## Discussion

LAMC1 is mainly expressed in the basement membrane and participates in several biological and pathological processes, including adhesion, invasion, and migration (Aumailley, 2013; Ke et al., 2013). In addition, LAMC1 may participate in some signaling pathways that affect cell proliferation and migration by activating intracellular downstream effectors (Ke et al., 2013). Interestingly, the *LAMC* gene family is also involved in kidney-related growth, development, and disease. An early report showed that LAMC1 interacts with nidogen to induce ureteric bud protrusion from the Wolffian duct in mammalian renal development (Willem et al., 2002). Besides, increased LAMC1 protein was also detected in glomerular basement membrane of kidney samples from chronic kidney disease (CKD) patients (Setty et al., 2012). A LAMC1 epitope fragment, LG1M, is a marker of remodeling and degradation of the glomerular and tubular basement membrane, and is related to disease progression and mortality in CKD (Holm Nielsen et al., 2018). Furthermore, a gene expression profile analysis identified the *LAMC1* gene as up-regulated in aggressive KIRC and as a candidate gene that differentiate aggressive from indolent KIRC phenotypes (Lane et al., 2009). In line with these early reports, it appears that high expression of *LAMC1* may be involved in the progression of kidney disease, including cancer. However, the correlation between *LAMC1* expression and the clinicopathological characteristics of RCC, as well as the prognostic significance of *LAMC1* expression for RCC have not been well studied.

In this study, bioinformatics analyses of high-throughput RNA sequencing data from TCGA revealed significantly increased *LAMC1* expression in RCC compared with the adjacent normal renal tissues, and the LAMC1 protein levels in RCC were also increased compared with the normal tissues based on tissue microarray data. Our results summarized for the first time the data on *LAMC1* expression in RCC. To explore the role of high expression of *LAMC1* in RCC, we further evaluated its effect on prognosis. According to the results of the KIRP survival analysis, patients with high *LAMC1* expression had worse survival than those with low expression, whereas in KIRC, high *LAMC1* expression predicted better survival. Univariate and multivariate Cox analysis of the TCGA database showed that *LAMC1* expression is a potential independent marker of poor prognosis in KIRP. Interestingly, KIRC showed the opposite result. The association between *LAMC1* expression and the clinical characteristics of RCC patients also confirmed this observation. These results suggested that *LAMC1* could be used as a marker of the cancer process to distinguish RCC patients from the normal persons; besides, the high expression of *LAMC1* in KIRP and KIRC has completely different clinicopathological significance and prognostic value.

Given that high *LAMC1* expression has significantly different prognostic value in KIRP and KIRC, we next tried to discover its potential regulatory mechanism. By analyzing the correlation between the *LAMC1* gene and immune cells, we found that *LAMC1* expression in KIRP was positively correlated with CD8^+^ T cells, myeloid dendritic cells and neutrophils. For KIRC, the *LAMC1* expression level showed a positive correlation with infiltrating levels of CD4^+^ T cells, macrophages, and neutrophils and a negative correlation with B cells. It is known that immune cells infiltrating the tumor, including macrophages, Treg cells, and CD8^+^ T cells can influence the outcome of RCC treatment (Desar et al., 2011; Cros et al., 2016; Zhu et al., 2019). Thus, the difference in immune cell types present in KIRP and KIRC probably affects the prognosis. In our study, high *LAMC1* expression in the cohort of KIRP patients with enriched Treg cells correlated with worse survival, whereas no such correlation was observed in the cohort of KIRP patients with fewer Treg cells. One of the mechanisms of tumor immune escape is that Treg cells produce immunosuppressive cytokines and receptors, which inhibit T cell activation and anti-tumor response (Sakaguchi et al., 2010; Speiser et al., 2016). The protective role of high levels of activated CD8^+^ T cells in various tumors have been proposed (Youngblood et al., 2017; Yao et al., 2018). In our study, we found that high *LAMC1* expression in the cohort of KIRC patients with enriched CD8^+^ T cells correlated with good survival, which was not significant for KIRP patients with high *LAMC1* expression; in contrast, high *LAMC1* expression in the cohort of KIRP patients with reduced CD8^+^ T cells correlated with poor survival, which was not significant for KIRC patients; on the opposite, *LAMC1* high expression in decreased CD8^+^ T cells cohort of KIRP showed a well OS but not in KIRC. This result suggests that *LAMC1* overexpression has different prognostic significance in KIRP and KIRC patient cohorts depending on CD8^+^ cell levels. Notably, KIRP patients with high *LAMC1* expression and reduced numbers of CD4^+^ memory T cells, Th1 cells, and Th2 cells had a worse prognosis, in contrast to similar cohorts in KIRC. These results indicate the potential functionality of assessing *LAMC1* expression and immune cell infiltration in the prognosis of RCC and treatment efficacy. Thus, KIRP and KIRC have different immune responses. The relationship between this complex immune cell infiltration and *LAMC1* expression affects the prognosis for RCC patients, but the underlined mechanism remains to be clarified, and the single-cell RNA sequencing may provide a potential solution to this problem.

Apart from the immune cells, immune factors also contribute to cancer progression. Using the TIMER database, we identified some of the immunoinhibitors and immunostimulators associated with *LAMC1* in KIRP and KIRC. The biological function of the insertion domain kinase receptor (*KDR*) is to regulate normal/pathological angiogenesis (Hoeben et al., 2004; Takahashi and Shibuya, 2005; Shibuya, 2010). Using the GSCALite database, we tested the correlation of *KDR* with prognosis in KIRC and KIRP, and showed that *KDR* is associated with poor survival in KIRP and better survival in KIRC. Our results are consistent with earlier reports suggesting that high *KDR* levels are significantly associated with poor prognosis for patients with KIRP (Kroeze et al., 2010). However, the positive association of *KDR* expression with survival in patients with KIRC requires further elucidation. Correlation analysis of expression between *LAMC1* and immunostimulators showed that *CD276* (*B7-H3*) and *NT5E* had a higher correlation with KIRP and KIRC. As a member of the B7 family of immunoregulatory ligands, *CD276* (*B7-H3*) plays a role in regulating the immune response (Picarda et al., 2016). High expression of B7-H3 protein correlates with poor outcome in patients with various types of cancer. We also observed that *CD276* is significantly associated with the poor prognosis of two kinds of RCC. Ecto-5′-nucleotidase (*NT5E/CD73*) mediates the sequential dephosphorylation of extracellular ATP to adenosine (Zimmermann, 1992). Increased signaling initiated by adenosine promotes the proliferation of Treg cells, the accumulation of intracellular cAMP, and the differentiation of tumor-associated macrophages, thereby reducing the anti-tumor immune response (Vigano et al., 2019). The correlation between *LAMC1* expression and these molecules suggests a possible mechanism, signaling pathway, and prognostic value for *LAMC1* in tumor immunity. The current study also showed that tumor neoantigens appearance and TMB have no or very weak association with KIRP and KIRC; we therefore focused on the immune scores, stromal scores and estimate scores in KIRP and KIRC. However, only the results of stroma scores assessment showed KIRC to be moderately positive. These results suggested that the high expression of *LAMC1* in KIRC may be accompanied by a better immune microenvironment. The above results may help to explain the correlation between high *LAMC1* gene expression and the better prognosis in KIRC.

DNA methylation is one of multiple epigenetic marks that regulate gene expression in cells (Ehrlich, 2002). Hypomethylation of the gene body leads to the high expression of oncogenes (Yang et al., 2014). Our study found that hypomethylation of *LAMC1* in two kinds of RCC is related to high expression of the *LAMC1* gene. In the present work, we not only confirmed the hypomethylation of *LAMC1* in KIRC suggested by others (Wu et al., 2018), but additionally found the hypomethylation in the *LAMC1* gene in KIRP. However, *LAMC1* hypomethylation in KIRP and KIRC weakly correlates with prognosis in cancer patients. Thus, although *LAMC1* hypomethylation in KIRP and KIRC is associated with high *LAMC1* expression, alone, it does not contribute significantly to the prognosis of RCC patients. Copy number variations (CNV) influences gene expression in carcinogenesis (Hudler, 2012). In our study, we found that higher CNV values correlated with lower survival in both KIRP and KIRC. Therefore, the CNV of the *LAMC1* gene can be used as a prognostic tool in KIRP and KIRC.

To further evaluate the role of *LAMC1* in KIRP and KIRC, we performed Gene Set Enrichment Analysis (GSEA) using TCGA data. GSEA analysis showed that genes involved in adherens junctions, extracellular matrix receptor interaction, MAPK signaling pathway, TGF-β signaling pathway, and Wnt signaling pathway were differentially associated with the *LAMC1* high expression phenotype. In addition, the mTOR pathway (Motzer et al., 2008) and the VEGF signaling pathway (Turner, 2004; Yildiz et al., 2004) involved in the pathogenesis of renal cancer were also enriched in our current study (data not shown). The results of using two major processes to examine groups of genes involved in common biological activities, Gene Ontology (GO) and KEGG enrichment analyses, using the Metascape portal to identify *LAMC1*-interacting genes, were similar to those of the GSEA analysis. Finally, drug sensitivity analysis revealed that cases with high *LAMC1* expression were resistant to most drugs or small molecules in the GDSC database. These results suggested that expression of *LAMC1* is a potential biomarker for drug screening and might provide a basis for drug-targeted therapy.

In conclusion, we have explored the expression, prognosis, and potential carcinogenic mechanism of *LAMC1* in KIRP and KIRC patients. Enhanced expression of *LAMC1* indicates a poor prognosis in KIRP and a better prognosis in KIRC. These opposite prognostic features of *LAMC1* overexpression in the two types of renal carcinoma may be related to different tumor immune microenvironments and immunomodulator-associated molecules. The results of our study will help clinicians to assess the prognosis and guide treatment of patients with KIRP and KIRC. However, future analysis of an independent patient cohort based on other data sources and experimental validation of the biological significance of *LAMC1* expression in RCC is needed.

## Data Availability

The datasets presented in this study can be found in online repositories. The names of the repository/repositories and accession number(s) can be found in the article/Supplementary Material.
