# Supplementary material for: Comprehensive analysis of LAMC1 expression and prognostic value in kidney renal papillary cell carcinoma and clear cell carcinoma
Source: Front Mol Biosci. 2022 Sep 16;9:988777. doi: 10.3389/fmolb.2022.988777 (PMC9523316; doi:10.3389/fmolb.2022.988777)
Supplement: Supplementary file 3 [file DataSheet4.PDF]

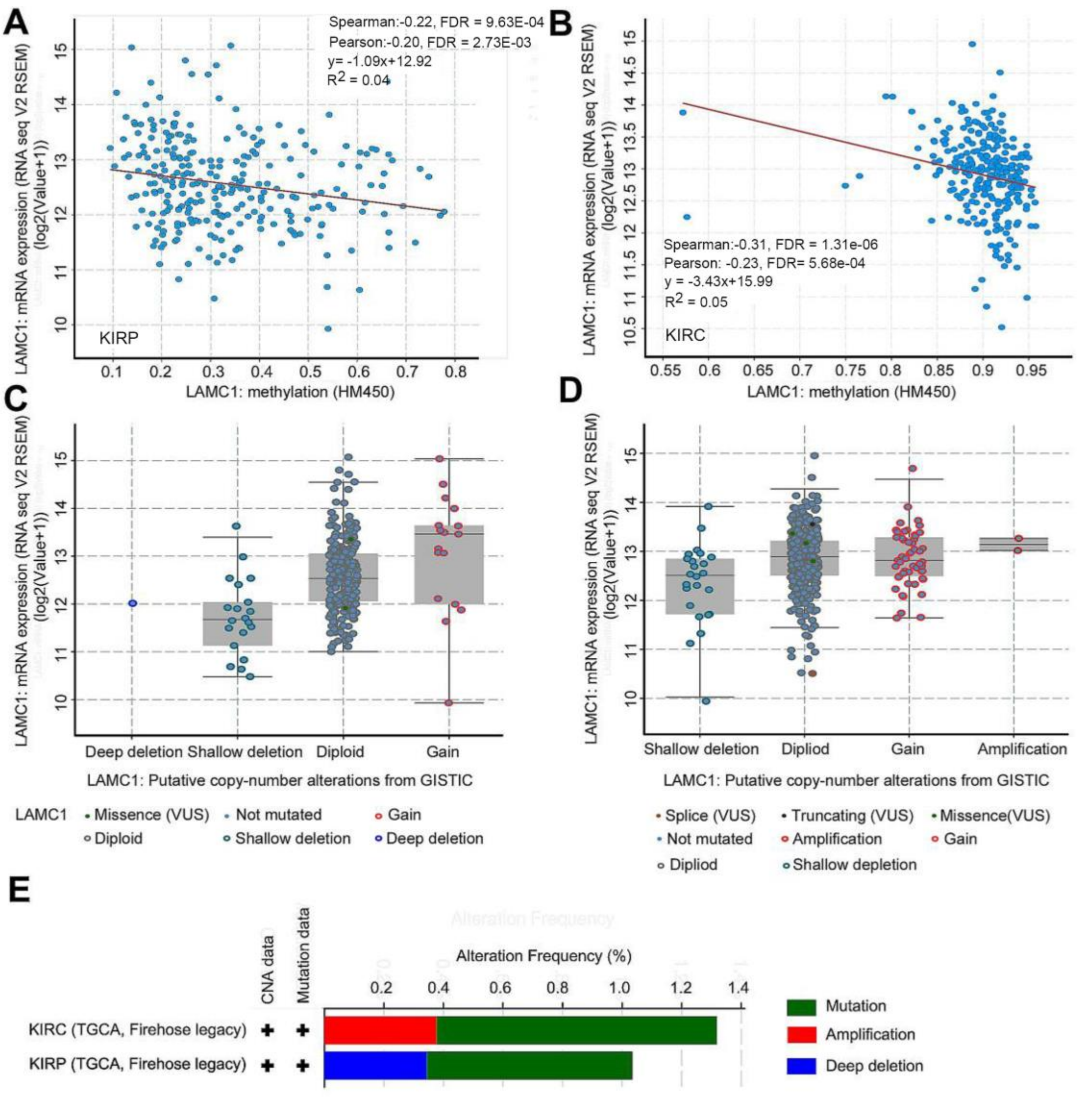

Supplementary **Figure S4**. The methylation of *LAMC1* gene, copy number variation (CNV) and mutation in KIRP an d KIRC. (A, B) The correlation between *LAMC1* gene methylation and the expression level in KIR P (A) and KIR C (B). (C, D) The *LAMC1* mRNA expression levels depending on the CNV values of *LAMC1* in KIRP (C) and KIRC (D). (E) Frequencies of *LAMC1* mutations and copy number alterations (CNA) in KIRC and KIRP. Diploid: two alleles present; Gain, Low-level gene amplification event; Amplification, high-level gene amplification event; Shallow Deletion, mild deletion of gene copy number; Deep Deletion, Deep deletion of gene copy number
