# Supplementary material for: Comprehensive analysis of LAMC1 expression and prognostic value in kidney renal papillary cell carcinoma and clear cell carcinoma
Source: Front Mol Biosci. 2022 Sep 16;9:988777. doi: 10.3389/fmolb.2022.988777 (PMC9523316; doi:10.3389/fmolb.2022.988777)
Supplement: Supplementary file 5 [file DataSheet3.PDF]

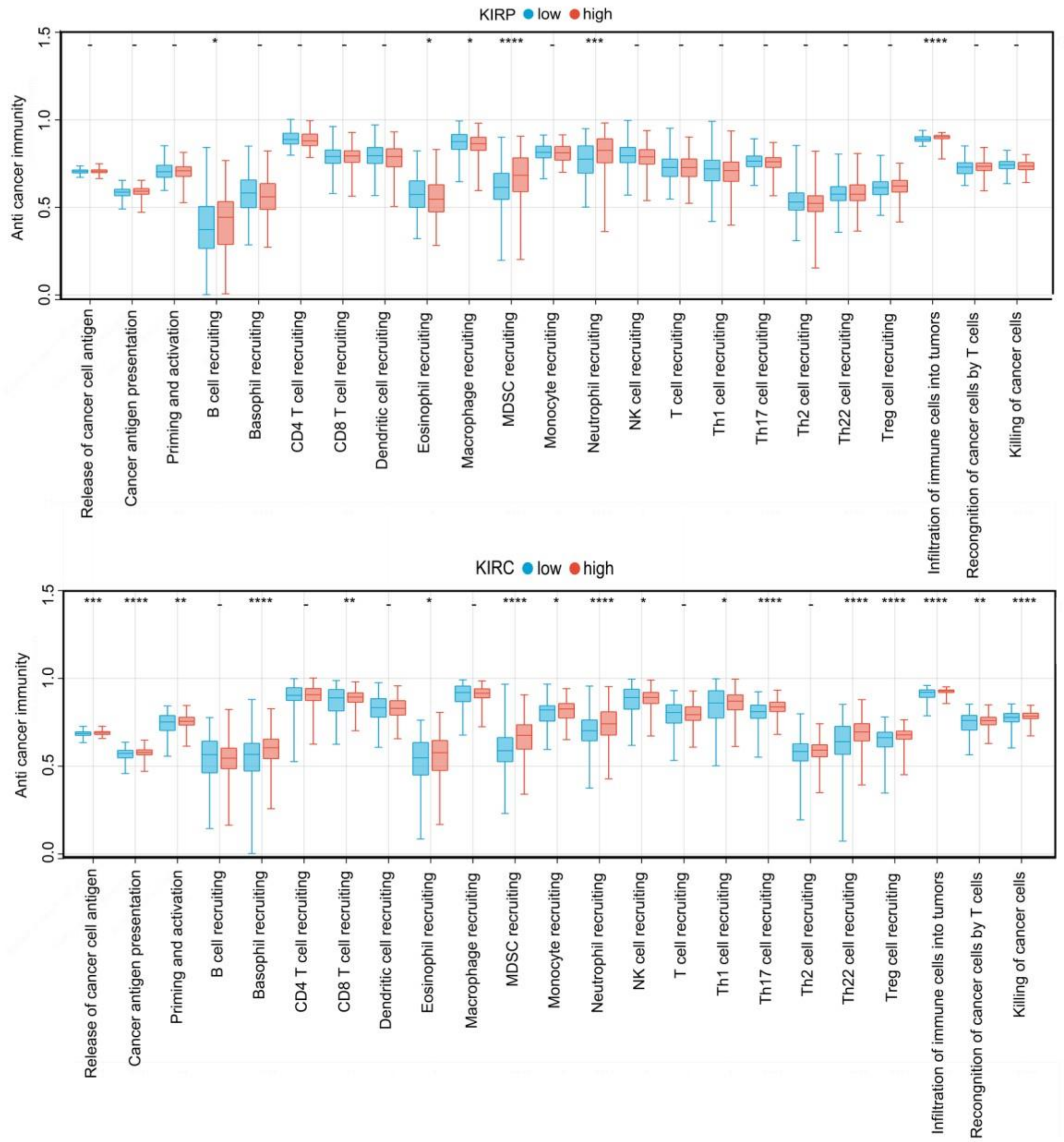

Supplementary **Figure S3**. Immunoactivity of the Cancer-Immunity Cycle in high/low *LAMC1* expression groups in KIRP and KIRC using ssGSEA algorithm with R package. These results were presented with boxplot with the assistance of online web-Sangerbox 3.0 (http://vip.sangerbox.com/home.html) with Student's *t*-test. The Cancer-Immunity Cycle consists of seven steps: release of cancer cell antigen (step1), cancer antigen presentation (step2), priming and activation (step3), B cell recruiting, Basophil recruiting, CD4 T cell recruiting, CD8 T cell recruiting, Dendritic cell recruiting, Eosinophil recruiting, Macrophage recruiting, MDSC recruiting, Monocyte recruiting, Neutrophil recruiting, NK cell recruiting T cell recruiting, Th1 cell recruiting, Th17 cell recruiting, Th2 cell recruiting, Th22 cell recruiting, Treg cell recruiting (step4), Infiltration of immune cells into tumors (step5), Recognition of cancer cells by T cells (step6), Killing of cancer cells (step7). The low and high expression of *LAMC1* was determined by the values of Median. *$P<0.05$, **$P<0.01$, ***$P<0.001$, ****$P<0.0001$.
