# Supplementary figures and images for: Comprehensive analysis of LAMC1 expression and prognostic value in kidney renal papillary cell carcinoma and clear cell carcinoma
Source: Front Mol Biosci. 2022 Sep 16;9:988777. doi: 10.3389/fmolb.2022.988777 (PMC9523316; doi:10.3389/fmolb.2022.988777)

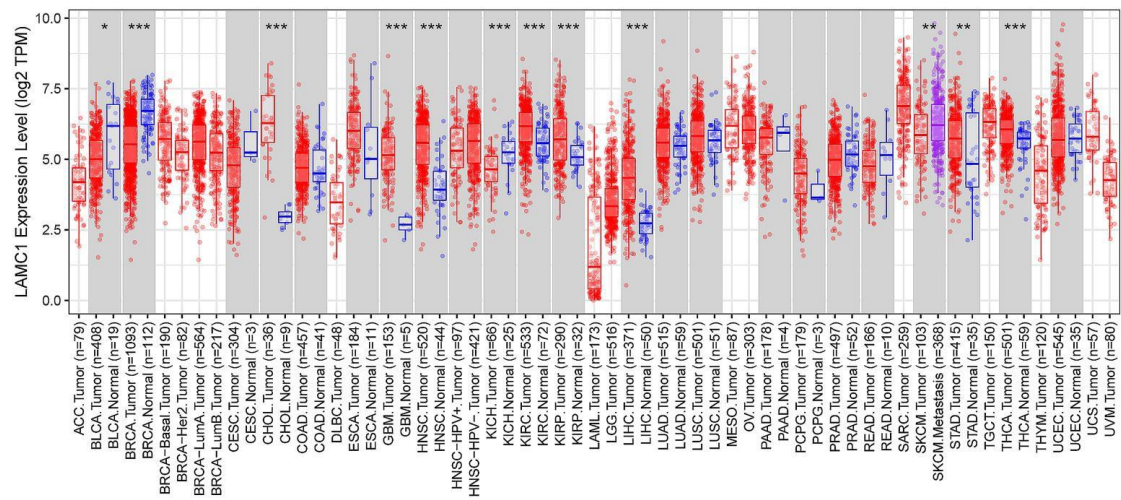

Supplement: Supplementary file 6 [file DataSheet1.PDF]
