# Supplementary material for: Comprehensive analysis of LAMC1 expression and prognostic value in kidney renal papillary cell carcinoma and clear cell carcinoma
Source: Front Mol Biosci. 2022 Sep 16;9:988777. doi: 10.3389/fmolb.2022.988777 (PMC9523316; doi:10.3389/fmolb.2022.988777)
Supplement: Supplementary file 1 [file DataSheet2.PDF]

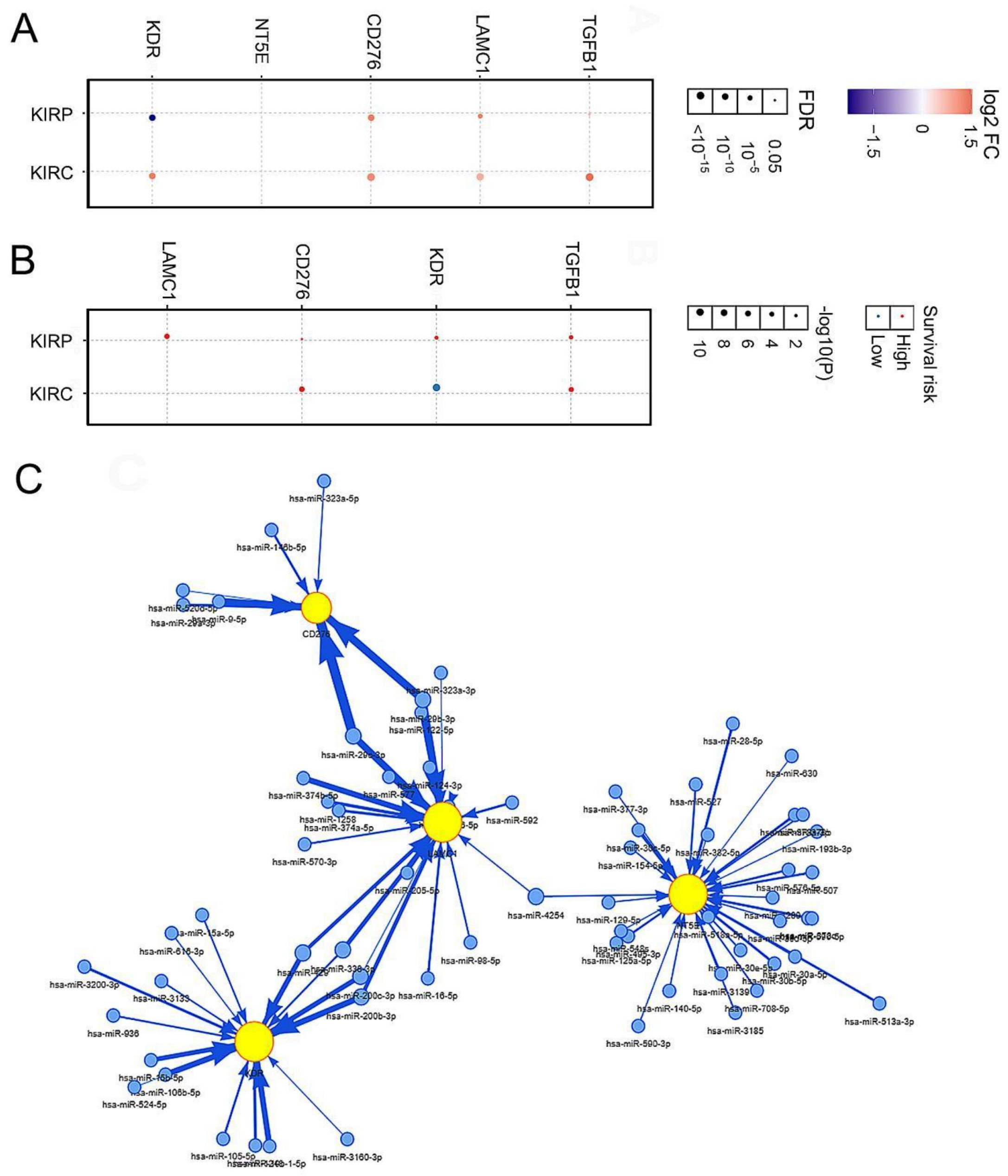

Supplementary **Figure S2**. Expression, prognosis and miRNA regulatory net for *LAMC1* and some immunomodulator-related genes in KIRP and KIRC. (A) Expression of *LAMC1*, *TGFB1*, *CD276*, *NT5E* and *KDR* in KIRP and KIRC. (B) The prognostic correlation of *TGFB1*, *CD276*, *NT5E* and *KDR* in KIRP and KIRC. (C) miRNA regulatory net for *TGFB1*, *CD276*, *NT5E* and *KDR* in RCC.
